# Sources of trust in the healthcare context. A multilevel relationship with work engagement and organisational outcomes

**DOI:** 10.3389/fpsyg.2024.1438872

**Published:** 2024-09-24

**Authors:** Juana Olvera, Hedy Acosta-Antognoni, Susana Llorens, Marisa Salanova

**Affiliations:** ^1^WANT Research Team, Department of Social Psychology, Universitat Jaume I, Castellón, Spain; ^2^Faculty of Psychology, Universidad de Talca, Talca, Chile

**Keywords:** horizontal trust, organisational resources, organisational outcomes, multilevel, healthcare sector

## Abstract

**Introduction:**

The present study analysed the mediating role of interactional justice and horizontal trust between transformational leadership and organisational outcomes (i.e., job performance and service quality) at the work team level and the cross-level relationship of team horizontal trust with job performance at the individual level, controlling for work engagement based on the HERO Model.

**Methods:**

Through structural equations and hierarchical linear models, the proposed hypotheses were addressed. The sample corresponds to 1,638 workers grouped into 109 work teams belonging to 28 hospitals in Spain.

**Results:**

First, Structural Equation Modelling analysis revealed that, as expected, at the team level, interactional justice and horizontal trust mediate positive and significantly the relationship between transformational leadership and organisational outcomes (i.e., job performance and service quality). Secondly, the results of the Linear Hierarchical Models showed a positive relationship between work engagement and individual level performance. Finally, the multilevel analysis revealed that horizontal trust at the team level is positively related to work engagement at individual level; however, there is no cross-level relationship between horizontal trust at the team level and individual performance controlled by work engagement.

**Discussion:**

Horizontal trust, at the team level, is positioned as a mediating variable between resources and organisational outcomes. Furthermore, it proves to be a key cross-level element for generating work engagement and job performance. The theoretical and practical implications of the study based on the HERO Model are discussed.

## 1 Introduction

Extensive research highlights the importance of trust operating at different levels in organisational contexts (Fulmer and Gelfand, 2012). Organisational trust enables workers to develop their strengths and, as such, has a positive influence on work well-being and organisational outcomes because, far from being just a psychological state, it fosters positive behaviours such as job performance, for example (Costa and Anderson, 2011; Salanova et al., 2019).

Over the years, organisations have evolved towards more horizontal and team-centred models and structures, with different approaches and more collaborative forms of management (Costa, 2003; Costa and Anderson, 2011). Nowadays, organisations are characterised by their multilevel structure so, in order to broaden the field of knowledge regarding organisational trust, it is important consider it from the different levels of analysis, i.e., at the individual level, at the work team level and at the organisational level (Fulmer and Gelfand, 2012; Puusa and Tolvanen, 2006).

In this line, the healthcare organisation, with a multilevel structure, is precisely characterised by its great complexity, highlighting - in all the strata involved - the multidisciplinary and multicultural nature of the organisation in which the work team has a particularly important value (Fernández and Mosquero, 2012; Olvera et al., 2017).

In the healthcare organisations’ work environments, trust is a crucial element in determining work well-being, worker performance, service quality and commitment to the organisation (Spence Laschinger et al., 2001; Salanova et al., 2019; Salanova et al., 2012a).

In addition, organisational trust is considered as a mediating variable between resources (i.e., transformational leadership, organisational justice) and organisational outcomes (i.e., performance, service quality) (Salanova et al., 2019; Salanova et al., 2021). Likewise, Fulmer and Gelfand (2012) show how research on organisational trust has been mostly focused on studies at the individual level, focusing to a lesser extent on the team and organisational levels.

Therefore, the present study analyses the antecedents and consequences of organisational trust (i.e., horizontal trust) on two levels (i.e., work team level, individual level) within the healthcare context and based on the HERO Model (HEalthy & Resilient Organisations Model; Salanova et al., 2012b).

The literature has considered the importance of trust in the context of organisations. However, there is still no consensus on its definition (Salanova et al., 2019). In a systematic review, Fulmer and Gelfand (2012) reveal that, in most definitions of organisational trust, two key aspects are distinguished: positive expectations and willingness to accept vulnerability.

Mayer et al. (1995) define trust as: “One party’s willingness to be vulnerable to another, based on the expectation that the other party will engage in conduct that is meaningful (beneficial) to him, regardless of his ability to control the other party” (p. 712).

For Rousseau et al. (1998), trust refers to “a psychological state comprising the intention to accept vulnerability based upon positive expectations of the intentions or behaviour of another” (p. 395). Subsequently, Tan and Lim (2009) define organisational trust as “the willingness of a person to be vulnerable to the actions of fellow co-workers, whose behaviour and actions that person cannot control” (p. 46).

Previous evidence shows how organisational practices and resources are important for the development of organisational trust and how this, in turn, has positive consequences on work well-being and organisational outcomes (Salanova et al., 2019). For example, Spence Laschinger et al. (2001), conducted research focusing on 412 nurses working in urban hospitals in a Canadian province. This study’s results support how professional empowerment is positively related to perceptions of trust that, in turn, improve job well-being in terms of satisfaction and organisational commitment. In the same vein, Guh et al. (2013) conducted a study in the university context involving 315 professors from different universities. Its results highlighted the mediating role of organisational trust between perceptions of organisational justice and organisational citizenship behaviours. Finally, Bartram and Casimir (2007) carried out a study of 150 workers in a call-centre company in Australia. The results obtained show the positive relationship between supervisors in transformational leadership roles and workers who trust in their leaders, which are positively related to overall job performance.

Along the same line, Salanova et al. (2012a) introduce the concept of HERO (HEalthy & Resilient Organisation) within the framework of Positive Occupational Psychology, defining it as “one that makes systematic, planned and proactive efforts to improve the processes and results of employees and the organisation” (p. 788). In addition, it is resilient because, when faced with adverse situations and circumstances, workers and work teams have the ability to face these critical situations, overcome them, maintain their functioning and emerge stronger (Salanova et al., 2019).

The HERO Model is a heuristic model, supported by empirical evidence, that enables the assessment and development of Healthy Organisations. The model identifies three key blocks or components that show interdependence among each other: (1) “resources (i.e., social and task resources) and healthy organisational practices,” referring to the development of strategies aimed at the organisation achieving its goals; (2) “healthy employees,” regarding the psychosocial well-being of workers and work teams (i.e., organisational trust, work engagement) and; (3) “healthy organisational outcomes” related, for example, to job performance and service quality (Salanova et al., 2014; Salanova et al., 2019; Salanova et al., 2012b).

In addition, the authors propose a methodology for the evaluation and development of HEROs that goes a step further, because: (1) it contemplates the combination of qualitative and quantitative methodology, (2) it takes into account the participation of the different key actors of the organisation (i.e., workers, supervisors, managers), (3) the measurement tools follow a group approach (i.e., team and organisational), (4) it allows for the analysis of the data within a group (i.e., work team) and organisational way, (5) it allows for the analysis of the data within the context of the HEROs, (3) the measurement tools have a group (i.e., work team) and organisational focus, (4) it allows the analysis of data at a collective level with a multilevel perspective and (5) it provides a macro view (i.e., individual, teams, organisational).

Therefore, based on the HERO Model, organisational trust is part of the second component called “healthy employees.” Likewise, the model contemplates two dimensions of organisational trust: *vertical trust*, referring to the degree to which workers trust the actions of their superiors or the organisation in which they work, and *horizontal trust* referring to the degree to which workers trust and enjoy the people they work with (Salanova et al., 2019; Salanova et al., 2012a).

On the other hand, Costa (2003) highlights the importance of delving deeper into the issue of trust, taking work teams into account, and shows how the trust of work team members (i.e., horizontal trust) was positively related to performance. Other research at the work team level shows, for example, how trust mediates the relationship between resources (i.e., teamwork) and team well-being (i.e., work engagement) (Acosta et al., 2019). Likewise, a meta-analysis of 55 empirical studies encompassing 3,671 work teams evidences the positive relationship between trust and team-level performance (Morrissette and Kisamore, 2020). Furthermore, it shows how team trust mediates the relationship between transformational leadership and team performance, as shown by a study conducted on a sample of 137 university students grouped into 44 teams (Boies et al., 2015). It seems that teams have become an essential characteristic for most organisations and trust has been recognised as a mediating construct between social resources and team-level results (Chou et al., 2013). On the other hand, researchers consider the need to study into organisational trust in greater depth, taking into account different levels of analysis (Fulmer and Gelfand, 2012; Guinot and Chiva, 2019).

Research has shown great interest in the study of transformational leadership within the context of organisations (Molero et al., 2007). Developed by Bass (1985), the transformational leader causes changes in the values, priorities and attitudes of their followers and motivates them, causing them to perform beyond their expectations, as well as reaching their maximum potential (Bass and Avolio, 1994). The transformational leader is a person who knows how to guide others towards an end that is perceived as shared and that, in addition, achieves the commitment of workers, work teams and the organisation (Salanova, 2008; Salanova et al., 2019).

A study conducted by Rafferty and Griffin (2004) identifies five dimensions of transformational leadership: (1) *vision*: expression of an idealised image of the future based on the organisation’s values; (2) *inspirational communication*: positive messages about the organisation and statements that generate confidence and motivation; (3) *intellectual stimulation*: promotes employees’ interest in thinking about the problem in new ways; (4) *support*: the leader cares about their employees and is aware of their needs; and (5) *personal recognition*: rewards and acknowledgement of effort and achievement. In the HERO Model (Salanova et al., 2012b), transformational leadership is contemplated in the first of its components, specifically the one referring to organisational resources (i.e., social resources).

For decades, research has focused on the direct relationships of transformational leadership on organisational outcomes (i.e., job performance). In recent years, however, research has evolved to focus on studying the psychological mechanisms mediating between these relationships, such as trust and organisational justice (Katou, 2015).

Organisational justice refers to the perceptions that workers have regarding what is fair within the organisation for which they work (Greenberg, 1987). The literature identifies three main dimensions of organisational justice. First, *distributive justice*, which refers to workers’ perception of fairness (i.e., equity) in relation to the distribution of rewards (Adams, 1996). Secondly, *procedural justice* refers to workers’ perception of fairness regarding the procedures through which decisions are made (Thibaut and Walker, 1975). Thirdly, Bies and Moag (1986) introduce the dimension of *interactional justice* regarding the workers’ perception of justice in interpersonal dealings in decision making, that is, the sensitivity with which decisions are communicated. In addition, Greenberg (1993) subdivides interactional justice into two dimensions that he identifies as *interpersonal justice*, which involves the degree to which workers perceive respectful and dignified treatment in relation to decision making and *informational justice*, which involves the degree of information provided by superiors in relation to the procedures adopted and the distribution of rewards (Dai and Xie, 2016).

Dirks and Ferrin (2002) conducted a meta-analysis, specifically based on 106 studies (27,103 participants) focused on organisational trust and its antecedents, exploring the primary relationships of trust with leadership and other constructs such as organisational justice. Among the meta-analysis results, it is evidenced that leadership (i.e., transformational leadership) is positively related to trust and organisational justice (i.e., interactional justice). It also evidences positive relationships between trust and outcomes, which indicates that future research should focus on analysing the mediation processes that may be involved. Another meta-analysis conducted by Akar (2018) with a total of 43 studies and a sample of 22,859 participants, focused on educational organisations, showed a high correlation between leadership, organisational justice and trust. In the HERO Model, interactional justice would be considered an element of the first component called “healthy organisational practices and resources”, specifically referring to social resources (i.e., informational justice, interpersonal justice) (Rodríguez et al., 2014; Salanova et al., 2019).

Based on the above, the following research hypothesis is proposed:

*Hypothesis 1*: At the team level, interactional justice (i.e., informational justice and interpersonal justice) fully mediates the relationship between transformational leadership and horizontal trust.

A healthy organisation invests in healthy practices and organisational resources (i.e., transformational leadership, interactional justice) that can foster work well-being and, thus, develop organisational outcomes such as the job performance of workers, work teams, the organisation and, also, the quality of service (Salanova et al., 2019). The job performance and service quality of work teams are part of the “healthy organisational outcomes” component of the HERO Model (Salanova et al., 2012a).

Job performance, an organisational outcome indicator, refers to “the actions and behaviours that are under the control of the individual and that contribute to the organisation’s objectives” (Rotundo and Sackett, 2002, p. 66). For Motowidlo and Keil (2012), it is defined as “an expected value for the organisation of discrete behavioural episodes that an individual carries out over a standard period of time” (p. 91). They highlight, therefore, performance as a behavioural property and an expected value for the organisation. At the team level, performance would refer to the behaviours, in this case, of work teams that contribute to the organisation’s objectives (Borman and Motowidlo, 1997).

Two dimensions of job performance are considered: *intra-role performance* refers to worker behaviours or conducts that are specific to the job, activities related to formal work, and *extra-role performance* refers to those behaviours or tasks that go beyond formal work, which are voluntary, positive and contribute to the technical base of the organisation (Goodman and Svyantek, 1999).

Service quality is another indicator of good performance that depends on the fit between the service received by the user and the expectations of the service by the organisation (i.e., healthcare organisation). In addition, service quality is a good indicator of the organisation’s optimal functioning and leads to the improvement of user satisfaction, workers, work teams and organisational performance (Mahdikhani and Yazdani, 2020; Salanova et al., 2019).

With respect to trust regarding organisational resources and outcomes, organisational trust at the team level (i.e., horizontal trust) is considered as a mediating variable between organisational resources (i.e., transformational leadership and interactional justice) and organisational outcomes (i.e., job performance and quality of service). Proof of this is provided by research such as the study conducted by Mahdikhani and Yazdani (2020) on the mediating role of interpersonal trust between professional leadership and team performance and service quality. On the other hand, a study by Salanova et al. (2021) conducted with a sample of 890 workers from 177 work teams and supervisors of those teams shows that, at the team level, horizontal trust mediates the relationship between task and social resources and the performance of teams as assessed by supervisors. Furthermore, a meta-analysis by De Jong et al. (2016) on horizontal trust conducted with 112 studies that contemplated a sample of 7,763 teams, evidenced the positive impact of horizontal trust on job performance at the work team level and, in addition, pointed out the need to research horizontal trust further. Finally, Yorulmaz et al. (2021) conducted a meta-analysis including 34 studies with a total sample of 17,271 participants on the relationship between the perceptions of justice and the organisational trust of teachers, whose results evidence the positive relationship between these variables.

Therefore, in relation to the above, the second study hypothesis is proposed:

*Hypothesis 2*: At the team level, interactional justice and horizontal trust fully mediate the relationship between transformational leadership and organisational outcomes (i.e., intra-role performance, extra-role performance, and service quality).

Work engagement is defined as “as a positive fulfilling work-related state of mind characterised by vigour, dedication and absorption” (Schaufeli et al., 2002, p. 74), where *vigour* refers to high levels of energy and mental resistance at work; *dedication*, to being strongly involved and identified in one’s work with manifestation of enthusiasm, pride and challenge and; *absorption*, referred to the state of total concentration and enjoyment of the tasks being carried out, with the feeling that time “goes by very fast” (Acosta et al., 2011; Salanova et al., 2019; Salanova et al., 2012b).

In the HERO Model, work engagement is part of the “healthy employees” component. Research has shown how work engagement has a positive impact on organisational outcomes, such as job performance and service quality (Hernández et al., 2014; Llorens et al., 2007; Torrente et al., 2012). In addition, studies place organisational trust as an antecedent of work engagement (Acosta et al., 2011; Acosta et al., 2015).

So far, there is little research to explain the cross-level effects of trust at the work team level as a mediating variable, between the antecedents and consequents of trust (Salanova et al., 2021). Therefore, the following study hypotheses are proposed in an exploratory manner:

*Hypothesis 3*: At the individual level, work engagement is positively related to job performance.

*Hypothesis 4*: Horizontal trust at the team level has a positive cross-level relationship with workers’ performance, controlled by work engagement at the individual level.

**Figure 1:**
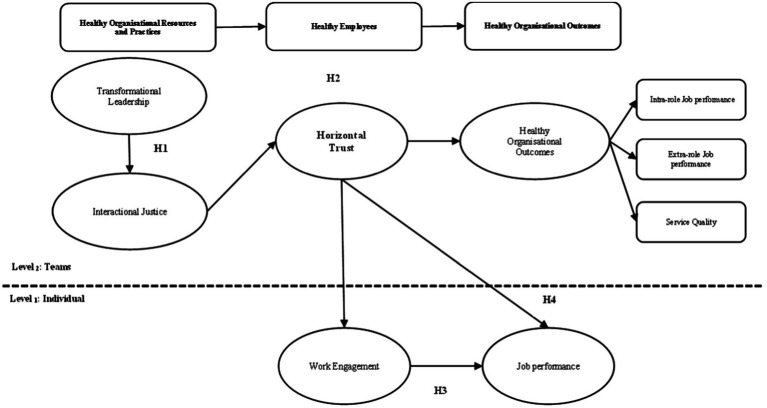
Research model (*N*_1_ = 1,638/*N*_2_ = 109).

Figure 1 shows the research model with study variables at different levels of analysis, as well as the study hypotheses.

## 2 Method

### 2.1 Participants and procedure

The sample is composed of 1,638 workers grouped into 109 work teams belonging to 28 hospitals in Spain. It is a representative sample with a margin of error of 0.03 for a confidence level of 95%. The mean age of the workers who participated in the study was 44 years (SD = 11), 79% of which were women. With regards to employment ties, 69% of the professionals who participated in the study had a permanent employment contract and the mean length of service within the organisation was 5.45 years (SD = 1.36). Finally, the average team size was 15 members (SD = 7.71).

According to McCarthy (1992), the inclusion criterion for the sample was only those professionals whose seniority in the company was greater than or equal to 6 months as, in this way, the professionals would have overcome the first stages of the labour socialization process and, as such, the response to the questionnaires would be as close to reality as possible.

The research focuses on work teams, made up of health centre professionals who share responsibilities, and are led by a supervisor responsible for the team (George, 1990).

The procedure followed the phases contemplated in the HERO methodology (Salanova et al., 2012a): (1) the management of the hospital centres was contacted to request their participation in the study; (2) following the acceptance by the management of the health centres, meetings were planned and held for the supervisors of the work teams, in which they were informed in detail of the aspects and procedure to be carried out, in addition to the ethical and legal aspects considered; and (3) the professionals of the teams who decided to participate voluntarily in the study completed an online questionnaire. Confidentiality was guaranteed, in compliance with current Personal Data Protection regulations.

### 2.2 Measures

#### 2.2.1 Team level

In these constructs, the referent was the team (i.e., “Now answer with your team in mind”).

##### 2.2.1.1 Transformational leadership

It was assessed using the five dimensions of the questionnaire by Rafferty and Griffin (2004) and adapted to Spanish by Salanova et al. (2012b). To answer the items, workers had to think about their immediate supervisor. The total scale is composed of 15 items distributed in five dimensions that are answered through a Likert-type scale from 0 (Strongly disagree) to 6 (Strongly agree): (1) Vision, three items (e.g., “*Understands perfectly what the objectives of the group are*”); (2) Inspirational communication, three items (e.g., “*Encourages us to see changes as situations full of opportunities*”); (3). Intellectual stimulation, three items (e.g., “*Has ideas that stimulate us to rethink some things we never thought about before*”); (4) Support, three items (e.g., “*Makes sure our interests are taken into account*”); and (5) Recognition, three items (e.g., “*Praises us when we do a better job than usual*”).

The scale has demonstrated a strong fit with the theoretical constructs, reflected in the Confirmatory Factor Analysis (CFA) fit indices: Comparative Fit Index (CFI) = 0.98; Tucker-Lewis Index (TLI) = 0.98; Root Mean Square Error Approximation (RMSEA) = 0.06; and Standardized Root Mean Square Residual (SRMR) = 0.02. Internal consistency by the McDonald Omega Coefficient (*ω*) showed the following indices: Vision (*ω*) = 0.91, IC 95% [0.90, 0.92], Inspirational communication (*ω* = 0.94, IC 95% [0.93, 0.95]), Intellectual stimulation (*ω*) = 0.94, IC 95% [0.93, 0.95], Support (*ω*) = 0.95, IC 95% [0.93, 0.96], and Personal Recognition (*ω*) = 0.97, IC 95% [0.96, 0.97]. In addition, Composite Reliability (CR) ranges from 0.91 to 0.97. Convergent validity through Average Variance Extracted (AVE) showed values between 0.77 and 0.91. Finally, discriminant validity is confirmed by observing the square root of the AVE, which exceeds the correlations between dimensions, thus indicating that each dimension is unique and measures distinct constructs.

##### 2.2.1.2 Organisational justice

It was evaluated through the organisational justice scale of Colquitt (2001) in its Spanish version (Díaz-Gracia et al., 2014) which was included in the HERO questionnaire (Salanova et al., 2012a) adapted to group level. Of the full scale that contemplates four dimensions, the dimensions of informational justice and interpersonal justice (i.e., interactional justice) were considered for this study, which are answered on a Likert-type scale from 0 (Strongly disagree) to 6 (Strongly agree): (1) informational justice, five items (e.g., “Our supervisor is sincere in communicating with us”), (2) interpersonal justice, four items (e.g., “Our supervisor treats us appropriately”).

The psychometric indicators of the scale reveal a structure well aligned with the theoretical model: CFI = 0.95, TLI = 0.93, RMSEA = 0.10 and SRMR = 0.04. Internal reliability by the McDonald Omega Coefficient *ω* showed the following indices = 0.94 (IC 95% [0.93, 0.96]) for Interpersonal Justice and *ω* = 0.96 (IC 95% [0.95, 0.96]) for Informational Justice. CR ranges from 0.94 and 0.95. Convergent validity through with AVE showed values: 0.84 for Interpersonal Justice and 0.82 for Informational Justice. Finally, discriminant validity was confirmed by observing the AVE, which was higher than the correlations between these dimensions.

##### 2.2.1.3 Horizontal trust

It was measured using McAllister’s questionnaire (1995) adapted and integrated into the HERO questionnaire at the group level (Salanova et al., 2012b). The scale consists of four items distributed in two observable indicators (with 2 items each) that are answered on a Likert-type scale from 0 (Strongly disagree) to 6 (Strongly agree). An example of an item is: “*The workers in my work team, we can talk freely with colleagues about possible difficulties we have at work*”.

This scale reflects a unidimensional structure, with fit indicators including a CFI = 0.96, TLI = 0.90, RMSEA = 0.20 and SRMR = 0.03. Internal consistency by McDonald Omega Coefficient *ω* was = 0.91 (IC 95% [0.90, 0.93]) and composite reliability = 0.91. Convergent validity, with an AVE = 0.60, is considered adequate.

##### 2.2.1.4 Healthy organisational outcomes

Three dimensions were considered in this section: Intra-role job performance, extra-role job performance and service quality. *Job performance* was assessed through an adaptation of the Goodman and Svyantek (1999) performance scale integrated in the HERO questionnaire (Salanova et al., 2012a). It consists of six items distributed in two dimensions that are answered on a Likert-type scale from 0 (Strongly disagree) to 6 (Strongly agree): (1) intra-role performance (3 items; e.g., “*The workers in my work team achieve the established performance criteria*”) and (2) extra-role performance (3 items; e.g., “*The workers in my work team perform functions that are not required but that improve the image of the organisation*”). *Service Quality* was assessed using the scale of Parasuraman et al. (1988) integrated in the HERO questionnaire (Salanova et al., 2012b). It consists of seven items that are answered on a Likert-type scale from 0 (Strongly disagree) to 6 (Strongly agree). An example item is “*We are able to put ourselves in the user’s shoes even when they have very specific needs*”.

The structure of these scales aligns well with the theoretical constructs, as indicated by a CFI = of 0.98, TLI = 0.97, RMSEA = 0.09 and SRMR = 0.04 for the job performance scale. Internal consistency by McDonald’s Omega Coefficient values of ω showed the following indices = 0.94 (IC 95% [0.93, 0.95]) and *ω* = 0.85 (IC 95% [0.83, 0.87]), respectively. CR ranges from 0.95 to 0.87. The AVE values (0.80 for inrol and 0.50 for extrarol) confirms convergent validity. Discriminant validity is confirmed by observing the square root of the AVE. The *Service Quality* showed the following indices: CFI = 0.95, TLI = 0.93, RMSEA = 0.10 and SRMR = 0.03; McDonald’s Omega *ω* = 0.93 (IC 95% [0.92, 0.94]), CR = 0.92 and AVE = 0.64.

#### 2.2.2 Individual level

In these constructs the referent was the workers at the individual level (i.e., “Now answer with your individual perceptions in mind”).

##### 2.2.2.1 Work engagement

This was assessed using the Spanish version of the UWES (*Utrecht Work Engagement Scale*) questionnaire for workers (Schaufeli et al., 2002; Schaufeli et al., 2006). It consists of 9 items, considered as a global measure of psychosocial well-being at work, whose response is based on a Likert-type scale from 0 (Strongly Disagree) to 6 (Strongly Agree) (e.g., “*I am strong and energetic in my work*”; “*I am enthusiastic about my work*”; “*I am focused on my work*”). Psychometric indicators suggest ω high internal consistency, with a CFI = 0.94, TLI = 0.93, RMSEA = 0.10 and SRMR = 0.03. Internal consistency by MacDonald’s Omega Coefficient ω was = 0.94 (IC 95% [0.93, 0.95]), CR = 0.93; AVE = 0.60.

##### 2.2.2.2 Individual job performance

It was assessed through an adaptation of Goodman and Svyantek’s (1999) performance scale integrated in the HERO questionnaire (Salanova et al., 2012a). It consists of six items, considered as a global measure of job performance whose response is based on a Likert-type scale from 0 (Strongly disagree) to 6 (Strongly agree): (e.g., “*The workers in my work team, we achieve the established performance criteria*”; “*The workers in my work team perform functions that are not required but improve the image of the organisation*”). The structure of these scales aligns well with the theoretical constructs, as indicated by fit indicators of CFI = 0.98, TLI = 0.97, RMSEA = 0.10 and SRMR = 0.036 for the job performance scale. They present values of McDonald’s Omega Coefficient, ω=0.93, Composite Reliability, CR = 0.90 and AVE = 0.62. The service quality scale showed the following indices: CFI = 0.95, TLI = 0.93, RMSEA = 0.10, SRMR = 0.03, McDonald’s Omega Coefficient, *ω* = 0.92 (IC 95% [0.91, 0.93]); CR = 0.92 and AVE = 0.64.

### 2.3 Data analysis

Using IBM-SPSS Statistics version 25.0, descriptive analyses (i.e., means, standard deviations, and correlations) were performed. The internal consistency of the scales was assessed using McDonald’s Omega Coefficient (*ω*) in R package. The McDonald’s Omega Coefficient provides a robust estimate of reliability in multifactor measurement models, as it does not assume tau equivalence. For the McDonald’s Omega Coefficient, values above 0.70 are considered acceptable, and values above 0.80 indicate good reliability (McDonald, 1999).

In addition, a Confirmatory Factor Analysis (CFA) was conducted to assess the psychometric properties of the instruments used in the sample. This analysis included the assessment of the convergent and discriminant validity of the scales, ensuring the robustness of the constructs measured.

To assess multicollinearity among predictors in the regression models, the Variance Inflation Factor (VIF) was calculated. A VIF value below 10 indicates no, or very low multicollinearity levels, while values above this threshold may signal issues that could compromise the stability of the regression coefficients. Additionally, the condition index was calculated as part of the collinearity diagnosis. A condition index above 15 suggests the presence of moderate multicollinearity, while values above 30 indicate severe collinearity (Neter et al., 1989).

The study is based on individual and team perceptions, Harman’s single-factor test (Podsakoff et al., 2003) was conducted to assess the presence of common method variance bias. To aggregate data at the team level, agreement indices (Chen et al., 2004) were calculated for the variable’s transformational leadership, interactional justice (including informational justice and interpersonal justice), horizontal trust, service quality, and job performance. The agreement index was calculated using Intraclass Correlation Coefficients (ICC1 and ICC2), with expected values above 0.12 and 0.60, respectively (Bliese, 2000; Glick, 1985). Finally, descriptive analyses (i.e., means, standard deviations, and correlations) were conducted on the aggregated dataset (*N* = 109).

Structural Equation Modelling (SEM) was conducted to study the team-level relationship of transformational leadership, interactional justice (i.e., interpersonal justice and informational justice), horizontal trust and healthy organisational outcomes (i.e., intra-role performance, extra-role performance and service quality) of teams. In this case, the Analysis of Moment Structures (AMOS vs. 23.0) program was used. To test the study hypothesis, two mediation models were compared: M_1_, the proposed model, in which interactional justice and horizontal trust at the team level fully mediate the relationship between transformational leadership and healthy organisational outcomes (i.e., intra-role performance, extra-role performance, and service quality) of teams; M_2_, a partial mediation model, in which a direct relationship between transformational leadership, interactional justice, and healthy organisational outcomes at the team level is also considered. Following Kline’s (1998) recommendations for cross-sectional studies, an alternative model was tested: M_A1_, where transformational leadership and interactional justice mediate the relationship between horizontal trust and healthy organisational outcomes (i.e., intra-role performance, extra-role performance and service quality) at the team level. The goodness of fit of the models was analysed by considering the following fit indices: Chi-square (*χ*^2^), the Chi-square ratio over the degrees of freedom (*χ*^2^/gl), and Root Mean Square Error Approximation, RMSEA. A *p* value associated with Chi-square greater than 0.05 indicates a good fit; likewise, the Chi-square ratio (*χ*^2^/gl) with values less than 2 indicate a good fit. RMSEA values less than 0.05 indicate very good fit; while values greater than 0.10 represent poor model fit (Browne and Cudeck, 1993). In addition, relative fit indexes were used, such as: Normed Fit Index, NFI; Tucker-Lewis Index, TLI or NNFI; Comparative Fit Index, CFI and the Incremental Fit Index, IFI. Values above 0.90 indicate a good fit (Hoyle, 1995). In addition, the Akaike Information Criterion (AIC; lower values of AIC indicate better fit) was calculated to compare non-nested competitive models (Marsh et al., 1996). It is considered that the lower the AIC value, the better the fit (Akaike, 1987). Also, relative fit indexes were used, such as: Normed Fit Index, NFI; Tucker-Lewis Index, TLI or NNFI; Comparative Fit Index, CFI; and the Incremental Fit Index, IFI. Values above 0.90 indicate a good fit (Hoyle, 1995). In addition, the Akaike Information Criterion (AIC; lower values of AIC indicate better fit) was calculated to compare non-nested competitive models (Marsh et al., 1996). It is considered that the lower the AIC value, the better the fit (Akaike, 1987).

Subsequently, using IBM-SPSS Statistics program version 25.0, the hypotheses referring to the cross-level relationships (i.e., Hypothesis 4) of this study were tested using Multilevel Models or Linear Hierarchical Models (Gavin and Hofmann, 2002). For this purpose, and as a first step in making this type of model, the Intraclass Correlation Coefficient (ICC) was calculated for the dependent variable, in this case, individual job performance. This was done using the so-called random-effects ANOVA model or Null Model. This model allows observing the variability within and between teams; therefore, it is an initial model that accepts that the intercepts vary randomly across teams (González-Romá, 2008). In other words, it evaluates the interdependence of job performance at the individual level, as an independent variable, which will allow us to see the percentage of the variable explained by a higher level (i.e., team level) (Hox, 2010).

Once the random-effects ANOVA or Null Model reports that there is sufficient explained variance of a higher level, we continue with the following multilevel models. Therefore, we continue with the following three models: (1) *Random regression coefficients model*, called Model 1, which provides us with information regarding Level 1 predictor variables, in this study considered at the individual level work engagement, taking into account the aggregate data structure and controlling for Level 2 covariances, i.e., team level; (2) *Intercepts as outcomes model*, called Model 2, which contemplates Level 2 and Level 1 variables as predictor variables of the intercept of the equation; that is, horizontal trust at the work team level (Level 2) and individual work engagement (Level 1); allowing to check the effect and covariances of Level 2 variables on Level 1 variables and, at the same time, controlling this effect and covariances by individual level variables and team level variables; and; (3) *Intercept and slope model as results*, called Model 3, in which individual work engagement and horizontal trust at the team level are taken into consideration, as predictor variables of Level 1 and Level 2, respectively.

It is important to consider that in Linear Hierarchical Models or Multilevel Models, the *χ*^2^ or chi-square estimator is considered an indicator of (good) fit of the models being tested. This assumes that, for each hypothesised model, the *χ*^2^ should decrease significantly (González-Romá, 2008).

Another relevant aspect is the centring of the study variables in the multilevel models. This means that: (1) in Model 1, where the variables are considered at the individual level (i.e., work engagement and individual job performance), they were centred at the group mean, because it allows for better interpretation when adjusting the estimator of the variance between teams (Hofmann et al., 2000); (2) in Model 2, the horizontal trust at the team level was centred at the grand mean, which allows decreasing the bias of multivariate regressions and having a better estimator (Bliese, 2000); in addition, centring to the grand mean allows us to reduce the correlation between the intercept and slope estimators across levels, that is, to reduce multicollinearity (Hofmann and Gavin, 1998) and; (3) in Model 3 (exploratory model), a new variable is calculated that considers the interaction between horizontal trust (at the team level) and work engagement (at the individual level) using the variables centred on the group (work engagement) and the grand mean (horizontal trust).

## 3 Results

### 3.1 Descriptive analysis and aggregation

Table 1 presents the results corresponding to the descriptive analyses (i.e., mean, standard deviation) and correlations of the study variables at the individual and team levels. The Intraclass Correlation Coefficients (ICC1 and ICC2) of the aggregate variables of the study are also included. Based on the assumption that these variables arise from shared perceptions of the team workers and, in order to justify the aggregation of the data at the level of work teams (Chen et al., 2004), the results obtained for the consistency indexes through the Intraclass Correlation Coefficients, ICC1, ranged between 0.51 and 0.90 and the ICC2 values between 0.80 and 0.91. Thus, these results support the aggregation of workers’ perceptions at the work team level.

**Table 1:** Descriptive analysis, correlations and McDonald’s Omega. (*N*_1_=1638 / *N*_2_=109).

*N*	Variables and levels	M	SD	ICC^1^	ICC^2^	1	2	3	4	5	6	7	8	9	10	11	12	13
1	TL-Vision (L_2_)	4.37	1.23	0.82	0.80	(0.90)	0.93***	0.91***	0.81***	0.85***	0.74***	0.86***	0.63***	0.56***	0.55***	0.52***	0.55***	0.58***
2	TL-Inspirational comunication (L_2_)	4.42	1.31	0.80	0.81	0.83***	(0.91)	0.94***	0.87***	0.92***	0.80***	0.90***	0.61***	0.59***	0.57***	0.53***	0.58***	0.60***
3	TL-Intellectual stimulation (L_2_)	4.09	1.32	0.75	0.81	0.80***	0.86***	(0.94)	0.80***	0.91***	0.76***	0.88***	0.65***	0.59***	0.60***	0.57***	0.56***	0.62***
4	TL-Support (L_2_)	4.31	1.39	0.81	0.83	0.75***	0.83***	0.79***	(0.95)	0.88***	0.86***	0.90***	0.67***	0.54***	0.61***	0.53***	0.57***	0.60***
5	TL-Personal recognition (L_2_)	4.35	1.45	0.79	0.80	0.72***	0.85***	0.80***	0.85***	(0.97)	0.80***	0.89***	0.66***	0.55***	0.59***	0.55***	0.56***	0.59***
6	OJ-Informational (L_2_)	4.41	1.29	0.64	0.82	0.75***	0.81***	0.79***	0.75***	0.81***	(0.96)	0.87***	0.57***	0.46***	0.55***	0.47***	0.46***	0.52***
7	OJ-Interpersonal (L_2_)	5.11	1.09	0.62	0.84	0.65***	0.71***	0.66***	0.81***	0.70***	0.75***	(0.94)	0.68***	0.60***	0.63***	0.60***	0.59***	0.64***
8	Horizontal trust (L_2_)	4.72	1.08	0.86	0.85	0.46***	0.49***	0.48***	0.49***	0.50***	0.43***	0.50***	(0.91)	0.73***	0.80***	0.72***	0.66***	0.80***
9	JP-In-role (L_2_)	4.92	0.91	0.56	0.80	0.47***	0.44***	0.42***	0.41***	0.41***	0.36***	0.45***	0.59***	(0.94)	0.83***	0.81***	0.69***	0.96***
10	JP-Extra-role (L_2_)	4.96	0.90	0.51	0.82	0.41***	0.42***	0.41***	0.39***	0.39***	0.35***	0.42***	0.59***	0.70***	(0.83)	0.84***	0.70***	0.95***
11	Quality of service (L_2_)	4.73	0.89	0.74	0.81	43***	0.44***	0.44***	0.41***	0.42***	0.35***	0.44***	0.56***	0.65***	0.69***	(0.93)	0.78***	0.86***
12	Work engagement (L_1_)	4.86	0.87	0.90	0.91	0.46***	0.46***	0.43***	0.41***	0.41***	0.34***	0.45***	0.52***	0.55***	0.55***	0.63***	(0.94)	0.73***
13	JP-Individual (L_1_)	4.94	0.84	0.59	0.88	0.47***	0.46***	0.45***	0.45***	0.43***	0.38***	0.47***	0.64***	0.92***	0.92***	0.73***	0.60***	(0.92)

***p < 0.001; M, mean; SD, standard deviation; ICC, intraclass correlation coefficient; TL, transformational leadership; OJ, organisational justice; JP, job performance; correlations at the individual level (under the diagonal) and at the team level (above the diagonal); McDonald’s Omega on the diagonal in parentheses; *L*_1_ = individual level and *L*_2_ = team level.

Regarding the reliability of the scales, the McDonald Omega Coefficient (*ω*) revealed high levels of internal consistency in all dimensions assessed, with *ω* values ranging from 0.85 to 0.97.

Additionally, results from the CFA conducted for each instrument used in the study indicated an adequate fit for their respective factor structures. The different models showed the following indices: CFI between 0.96 and 0.98, a TLI between 0.95 and 0.97, a RMSEA value between 0.05 and 0.07, and an SRMR between 0.02 and 0.04, supporting the adequacy structure of the constructs.

In terms of convergent validity, the Average Variance Extracted (AVE) values exceeded 0.50 for all dimensions, indicating that the scales capture a significant proportion of the variance of the constructs measured. Indeed, discriminant validity was confirmed by observing that the square root of the AVE of each dimension was higher than the correlations between the different dimensions, thus suggesting that each construct is distinct and measures a separate conceptual entity.

The results of the VIF showed acceptable indices, that ranges from to 1.41 to 7.06, suggesting that multicollinearity is not a significant problem in the models evaluated.

Additionally, the diagnosis of collinearity using the condition index revealed a maximum value of 31.69. The variance ratios also show that the dimensions Vision, Inspirational Communication, and Informational Justice (JINF) contribute significantly to this collinearity, with variance ratios of 0.25, 0.85 and 0.21, respectively.

On the other hand, and as expected, Harman’s test results show a poor fit to the data, *χ*^2^ (54) = 569.31, RMSEA = 0.29, CFI = 0.73, TLI = 0.67, IFI = 0.73. Therefore, the data indicate that the common variance bias does not pose a difficulty for the study.

Looking at the correlation analysis between the scales assessed at the team level, the results show that all dimensions of the transformational leadership scale, interactional justice (i.e., informational justice and interpersonal justice), horizontal trust and healthy organisational outcomes (i.e., intra-role performance, extra-role performance and service quality) correlate significantly and positively, where Pearson’s *r* values fluctuate between 0.92 and 0.35 (*p* < 0.001).

### 3.2 Model fitting: structural equation modelling

To carry out the Structural Equation Modelling (SEM) analyses, the aggregated team-level database (*N* = 109) was used. Specifically, four latent variables were used: (1) transformational leadership was composed of five indicators: vision, inspirational communication, intellectual stimulation, support and recognition; (2) interactional justice was composed of two indicators: informational justice and interpersonal justice; (3) horizontal trust was composed of two indicators with two items each; (4) healthy organisational outcomes was composed of three indicators: intra-role job performance, extra-role job performance and perceived quality of service.

As shown in Table 2, the results of the Structural Equation Models indicate that the proposed model (M_1_) offers a good fit to the data. On the other hand, in relation to the M_2_ model, there are no statistically significant differences (Diff M_1_ − M_2_ = 3.58(2), *p* = 0.17, ns), however, the direct relationship between transformational leadership and healthy organisational outcomes (i.e., intra-role performance, extra-role performance and service quality) is not significant (*β* = 0.74, *p* = 0.82). In addition, the relationship between interactional justice and healthy organisational outcomes (i.e., intra-role performance, extra-role performance, and service quality) is not significant (*β* = 0.21, *p* = 0.40). The results, therefore, indicate that, as predicted, M_1_ is a better model, showing double mediation.

**Table 2:** Fit indices for structural equation models (*N* = 109 work teams).

Models	*χ* ^2^	df	*p*	*χ*^2^/df	RMSEA	CFI	TLI	IFI	NFI	AIC	HOELTER 0.5	HOELTER 0.1	∆*χ*^2^	∆df	∆RMSEA	∆CFI	∆TLI	∆IFI	∆NFI	∆AIC
M_1_	100.05	49	0.000	2.04	0.098	0.97	0.97	0.97	0.95	180.00	72	81								
M_2_	96.42	47	0.000	2.05	0.099	0.98	0.96	0.98	0.95	182.42	72	82								
Diff. M_1_ − M_2_													3.58	2	0.01	0.01	0.01	0.01	0.00	2.42
M_A_	296.42	49	0.000	6.05	0.22	0.87	0.83	0.88	0.85	354.41	25	28								
Diff. M_1_ − M_A_													196.37	0	0.12	0.010	0.014	0.009	0.01	174.41

*χ*^2^, chi-square; df, degrees of freedom; *χ*^2^/df, absolute goodness of fit index; TLI, Tucker–Lewis index; IFI, incremental fit; CFI, comparative fit index; NFI, normed fit index; RMSEA, root mean square error of approximation; AIC, Akaike information criterion; Diff., difference.

Therefore, transformational leadership: (1) is positively and significantly related to interactional justice, *β* = 0.96, *p* < 0.001; *R*^2^ = 92%, (2) interactional justice is positively and significantly related to horizontal trust, *β* = 0.72, *p* < 0.001; *R*^2^ = 52% and (3) trust is positively and significantly related to healthy organisational outcomes (i.e., intra-role performance, extra-role performance and service quality), *β* = 0.86, *p* < 0.001; *R*^2^ = 74%.

As for the proposed alternative Model, M_A1_, where transformational leadership and interactional justice mediate the relationship between horizontal trust and healthy organisational outcomes (i.e., intra-role performance, extra-role performance and service quality) shows a “weaker” fit to the data compared to the other models.

### 3.3 Multilevel models

Table 3 shows the results of three Hierarchical Linear Models exploring the cross-level relationship between horizontal trust and individual job performance.

**Table 3:** Multilevel model: horizontal trust (team level) to job performance controlling for work engagement at the individual level. (*N*_1_ = 1,638/*N*_2_ = 109).

Parameters	Null model	Model 1	Model 2	Model 3
Intercept	4.92***	2.36***	4.94***	4.94***
Work engagement (WE)	–	0.53***	0.49***	0.50***
Horizontal trust (CH)	–	–	0.32***	0.31***
Interaction CH*WE	–	–	–	0.03 (*p* = 0.958)
*χ* ^2^	4558.63	3967.90	3210.50	3209.60
Δ*χ*^2^	–	590.73***	63.37***	2.13 (*p* = 0.54)
df	3	4	5	8

***p < 0.001.

The Null Model (ANOVA Model) indicates that 17% of the variance in individual job performance is explained by higher level variables, with a *χ*^2^ value of 4,558.63 (df = 3). This result justifies the continuation with the hypothesised models.

In Model 1 (random regression coefficients model), it is observed that work engagement at the individual level is positively and significantly related to individual job performance (*β* = 0.53, *p* < 0.001). This model significantly improves the fit compared to the Null Model, as indicated by the significant decrease in the *χ*^2^ value; *χ*^2^(1) = 3967.90, Δ*χ*^2^ = 590.73, *p* < 0.001.

Model 2 (intercept model as outcomes) includes horizontal trust and shows that this variable is also positively related to individual job performance (*β* = 0.32, *p* < 0.001), while the effect of work engagement remains significant (*β* = 0.49, *p* < 0.001). The inclusion of horizontal trust further improves the model, as evidenced by the significant reduction in the value of *χ*^2^; *χ*^2^(1) = 3210.50, Δ*χ*^2^ = 63.37, *p* < 0.001.

Finally, Model 3 (intercept model and slopes as outcome) introduces the interaction between horizontal trust and work engagement (HT*WE). However, this interaction is not significant (*β* = 0.03, *p* = 0.958), indicating that the relationship between work engagement and job performance does not vary significantly as a function of horizontal trust levels. Furthermore, the improvement in model fit is not significant compared to Model 2; *χ*^2^(3) = 3209.60, Δ*χ*^2^ = 2.13, *p* = 0.54.

**Figure 2:**
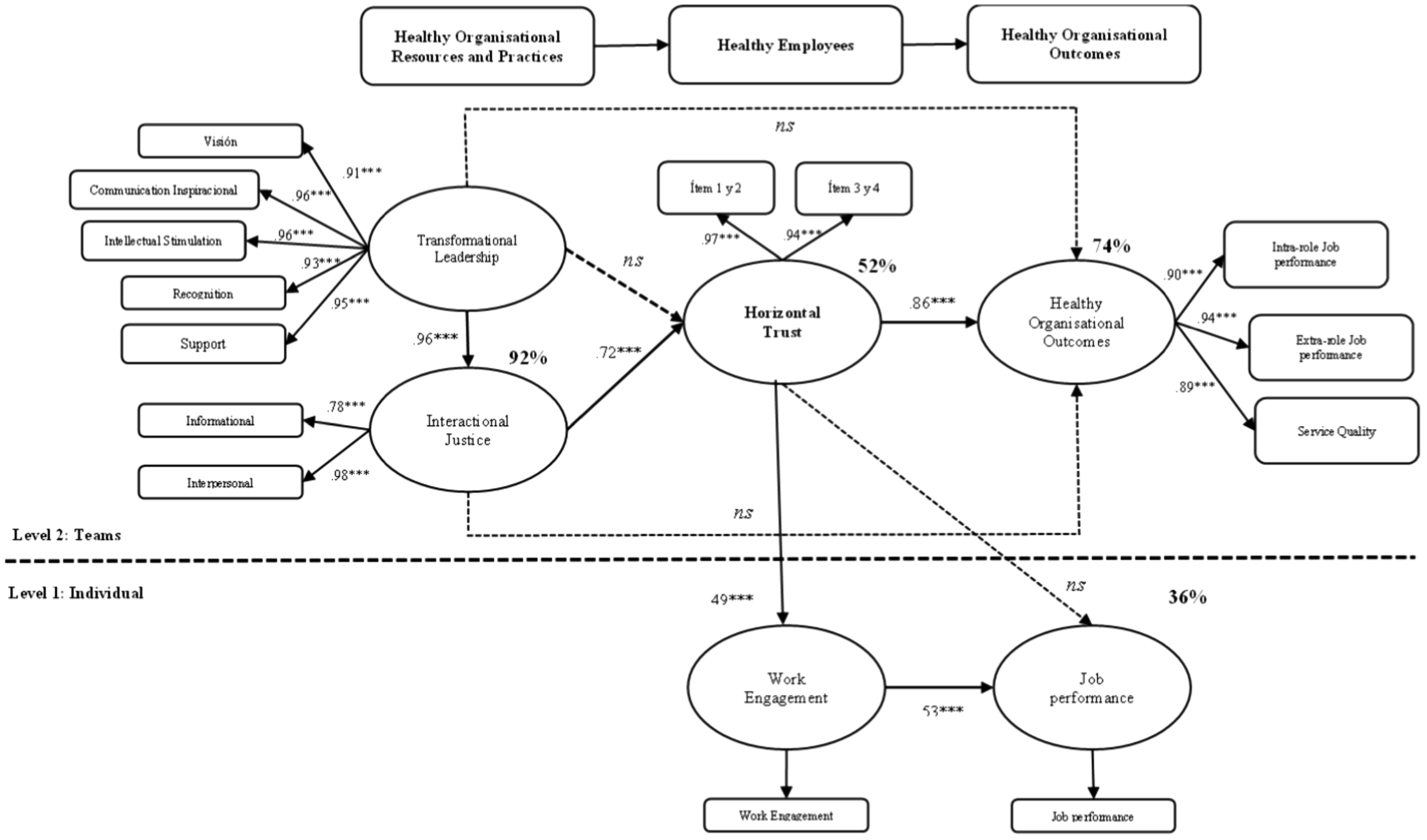
Final model (*N*_1_ = 1,638/*N*_2_ = 109).

These results confirm a positive and significant relationship between work engagement and job performance at individual level and the cross-level effect from horizontal trust to performance, where this interaction was not statistically significant. The final research model is graphically represented in Figure 2.

## 4 Discussion

The present study analysed the mediating role of interactional justice and horizontal trust between transformational leadership and organisational outcomes (i.e., job performance and service quality) at the work team level and the cross-level relationship of team horizontal trust with job performance at the individual level, controlling for work engagement, considering the HERO Model (Salanova et al., 2012b) as a reference and in the healthcare context.

It is worth noting that 76% of the sample participants were women, which aligns with the reality of many healthcare environments. This figure closely matches the World Health Organization (2019) report, which states that 70% of healthcare professionals are women. Therefore, the study sample offers a more accurate and contextualized perspective of the findings.

High correlations were observed between items from different measurement scales. Values obtained for the VIF were within acceptable limits, ranging from 1.41 to 7.06. In addition, the collinearity diagnosis via the condition index revealed a maximum value of 31.69. This index, primarily associated with the inspirational communication dimension of transformational leadership, indicates a high level of collinearity. Furthermore, the variance coefficients reveal that the vision and inspirational communication dimensions of transformational leadership, along with the informational justice dimension of interactional justice, significantly contribute to this collinearity, with variance coefficients of 0.25, 0.85, and 0.21, respectively. These findings suggest that, despite the Structural Equation Models (SEM) showing a good overall fit, collinearity among certain variables may have impacted the interpretation of relationships between constructs.

The SEM analyses, conducted using aggregated data at the team level, provide evidence for the following: (1) a full mediating role of interactional justice in the relationship between transformational leadership and horizontal trust at the team level, confirming our first hypothesis; (2) a mediation effect of interactional justice (i.e., informational and interpersonal justice) and horizontal trust between transformational leadership and organisational outcomes (i.e., intra-role performance, extra-role performance, and service quality) at the team level, which aligns with our second hypothesis. These results indicate that healthcare teams with transformational leaders as supervisors foster trust among team members. Moreover, trust perceptions increase when teams perceive that decision-making information and procedures are communicated fairly and with respect and dignity. This, in turn, enhances organisational outcomes, such as improved job performance and better service quality as perceived by healthcare teams. These findings are consistent with the work of Hernández et al. (2014) and Olvera et al. (2017), who highlighted trust as a key element between organisational resources, practices, and positive outcomes in line with the HERO model.

In addition, the Linear Hierarchical Models analysis showed a positive and significant relationship between work engagement and job performance at individual level, in line with our third hypothesis. This implies that workers who feel engaged in their roles are more energized, dedicated, and absorbed in their tasks, which enhances their motivation and, consequently, job performance (Christian et al., 2011; Lisbona et al., 2018; Motyka, 2018; Salanova et al., 2019; Schneider et al., 2017).

Additionally, horizontal trust at the team level is positively related to work engagement at individual level, suggesting that both intra-team trust and individual engagement are important for improving job performance. The inclusion of horizontal trust further improves the fit of the model. This finding mirror previous studies that have provided evidence on the positive relationship between trust and work engagement at both team and individual levels (Acosta et al., 2019; Lin, 2010).

However, the interaction between horizontal trust at the team level and individual job performance, when controlling for work engagement, was not statistically significant. This indicates that the relationship between work engagement and individual job performance does not significantly vary based on horizontal trust levels. Therefore, while both factors are important individually, they do not amplify each other in a way that would further impact job performance. This might suggest that interventions aimed at improving performance in healthcare settings should address intra-team trust and individual commitment as separate factors, without expecting their combination to produce a multiplicative effect.

### 4.1 Theoretical implications

The present study contributes to the research by providing evidence on the mediating role of horizontal trust at the team level between organisational resources (i.e., transformational leadership, interactional justice) and organisational outcomes (i.e., job performance and service quality), again supporting the HERO Model in the healthcare context.

According to the social exchange theory (Blau, 1964), organisational resources such as transformational leadership and interactional justice (i.e., interpersonal and informational) can foster and be sources that generate horizontal trust in teams, understood as “the willingness of a person to be vulnerable to the actions of fellow co-workers, whose behaviour and actions that person cannot control.” (Tan and Lim, 2009, p. 46); motivating attitudes and behaviours that improve results in terms of performance and quality of service. In this sense, when teams have transformational leaders, the existing interactions generate shared perceptions on the part of the team members fostering trust among them, which will improve performance - not only formal performance, but also performance that goes beyond what is stipulated by their job requirements and, in addition, improves the overall quality of service.

### 4.2 Practical implications

These results could be especially relevant for healthcare organisations, so that those responsible can develop positive psychological interventions (Salanova et al., 2013) (i.e., educational and training programs), considering an experimental design aimed at examining the efficacy of this intervention including control groups (Hernández-Sampieri et al., 2006). In this line, the design and implementation, for example, of program training in transformational leadership, emotion regulation, and character strengths among team leaders (i.e., supervisors) could be an adequate strategy that generate a positive impact.

### 4.3 Limitations and future investigations

While our study provides valuable insights on the effect of horizontal trust in the healthcare context, some limitations have been identified. Firstly, while the sample is cross-sectional, which limits our ability to examine both the antecedents and consequences of horizontal trust from a causal perspective, a multilevel approach was conducted given the specific characteristics of the healthcare organisation. Longitudinal research models should be considered in the future in order to understand if these effects are stable across different time intervals. Secondly, data collection was carried out using self-report questionnaires, which has inherent problems such as an unrealistic assumption that respondents can provide unbiased self-evaluations (Nisbett and Wilson, 1977; John and Robins, 1994). However, with the exception of individual work engagement and job performance scores, perceptions at the team level were considered for the multilevel analysis. In addition, Harman’s test was performed, whose indicators show that there is not an adequate fit to the data. This shows that the common variance bias in relation to health workers’ perceptions does not hinder the results of our study.

Thirdly, the composition of the sample may represent another limitation. Although the sample is representative of the health sector, with 76% female participants, this predominance of participants of one sex may limit the generalisability of the results to populations with a different sex distribution. However, given that the health sector is largely composed of women, the sample accurately reflects this reality. Although this study considers a convenience sample, the fact that the professionals participating in the study belonged to different hospital centres nationwide may provide a more comprehensive view of the reality. Fourthly, the presence of high correlations between some variables may indicate collinearity. While the VIF analysis showed that the values remained within acceptable ranges, the condition index revealed a high value, suggesting the presence of significant collinearity in certain variables such as the “communication” dimension of the transformational leadership construct. In this sense, future research should consider strategies such as reviewing and refining the items that make up these scales in order to mitigate these effects. Given that healthcare organisations are characterised by great complexity and specificity, it may be necessary to develop instruments specifically adapted to this context. The instruments currently used, although robust, may not fully capture the particularities of organisational dynamics and interactions in this context.

Ultimately, our results demonstrate the crucial role of horizontal trust in mediating the processes that enable healthcare organisations to generate good workplaces and organisational outcomes at the team and individual levels.

## Data Availability

The raw data supporting the conclusions of this article will be made available by the authors, without undue reservation.
